# Just Do It: Action-Dependent Learning Allows Sensory Prediction

**DOI:** 10.1371/journal.pone.0026020

**Published:** 2011-10-05

**Authors:** Itai Novick, Eilon Vaadia

**Affiliations:** The Edmond and Lily Safra Center for Brain Sciences, Department of Medical Neurobiology, Hadassah Medical School, The Hebrew University of Jerusalem, Jerusalem, Israel; University of Texas at San Antonio, United States of America

## Abstract

Sensory-motor learning is commonly considered as a mapping process, whereby sensory information is transformed into the motor commands that drive actions. However, this directional mapping, from inputs to outputs, is part of a loop; sensory stimuli cause actions and vice versa. Here, we explore whether actions affect the understanding of the sensory input that they cause. Using a visuo-motor task in humans, we demonstrate two types of learning-related behavioral effects. Stimulus-dependent effects reflect stimulus-response learning, while action-dependent effects reflect a distinct learning component, allowing the brain to predict the forthcoming sensory outcome of actions. Together, the stimulus-dependent and the action-dependent learning components allow the brain to construct a complete internal representation of the sensory-motor loop.

## Introduction

Meaningful interactions with the environment are based on sensory-motor learning. In order to turn a page in a journal, for example, one should first learn how to translate relevant sensory information (e.g., the location of the page) into the appropriate action. As one gains statistical knowledge of the outcome of previous actions (e.g., hand movement while gripping the page), one can also learn to estimate how future actions would affect subsequent sensory information (e.g., the next location of the page).

**Figure 1:**
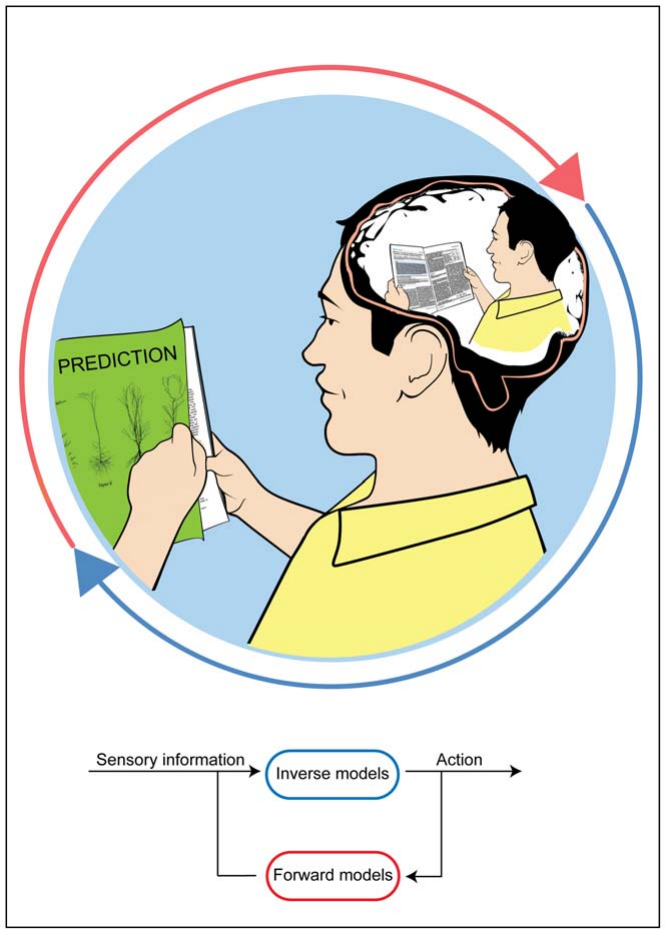
Circular sensory-motor information flow enables two types of learning. Mapping of sensations to actions (given the current sensory input, how should I turn a page?) and mapping of actions to sensations (given the current motor command to the hand, what should I see?).

Current opinion holds that the brain utilizes internal models of the relationship between the body and the world [1]–[8]. Inverse models allow transforming of sensory inputs and desired goals into motor commands (Figure 1, blue). Forward models act in the opposite direction: they allow the brain to predict the sensory outcome of actions (Figure 1, red).

A combination of inverse and forward models generates a loop (Figure 1, bottom): sensory information causes actions, and these actions subsequently affect sensory information. Traditionally, however, experimental research on sensory-motor learning has studied the loop from a single direction, investigating how subjects change their motor response to a given stimulus (i.e., how the sensory input leads to the motor output).

Here, we aimed to focus on the effects of motor actions on the understanding of their sensory consequences. To that end, we re-examined the classic “visuo-motor rotation” task, using a design that allowed us to assess the individual contributions of this learning component.

## Results

**Figure 2:**
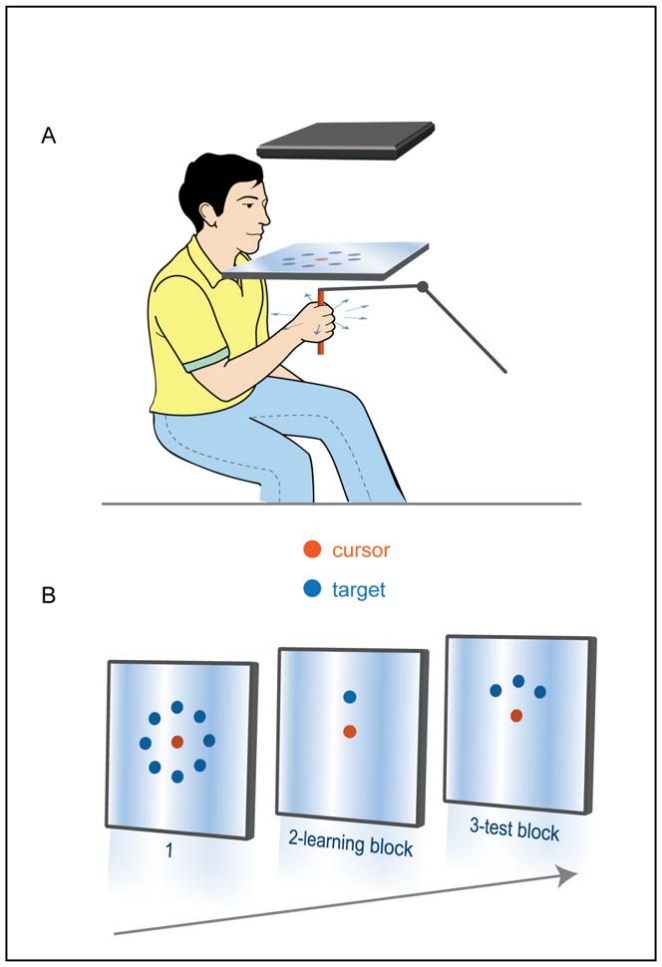
The experimental design. (**A**) Side-view of the experimental setup. Only one of the eight drawn targets appeared at a time. (**B**) Possible target locations in the three blocks of the task.

During the experiment, subjects sat in front of a workstation (Figure 2A) and grasped the handle of a lightweight robotic arm. Sphere cursor and targets were projected onto a mirror, placed horizontally above the subjects' shoulders. The subjects controlled the cursor by moving the robotic arm. They could not see their hand or the robotic arm while performing the task.

The experiment consisted of three blocks, presented sequentially in a single session (Figure 2B). During the first block, the subjects were trained to move the cursor from a central position to a target at one of eight locations. Targets were radially arrayed around the center (45° apart) and were presented in a pseudorandom order. The 3D effect was adjusted to display the location of the cursor at the location of the hand in space.

**Figure 3:**
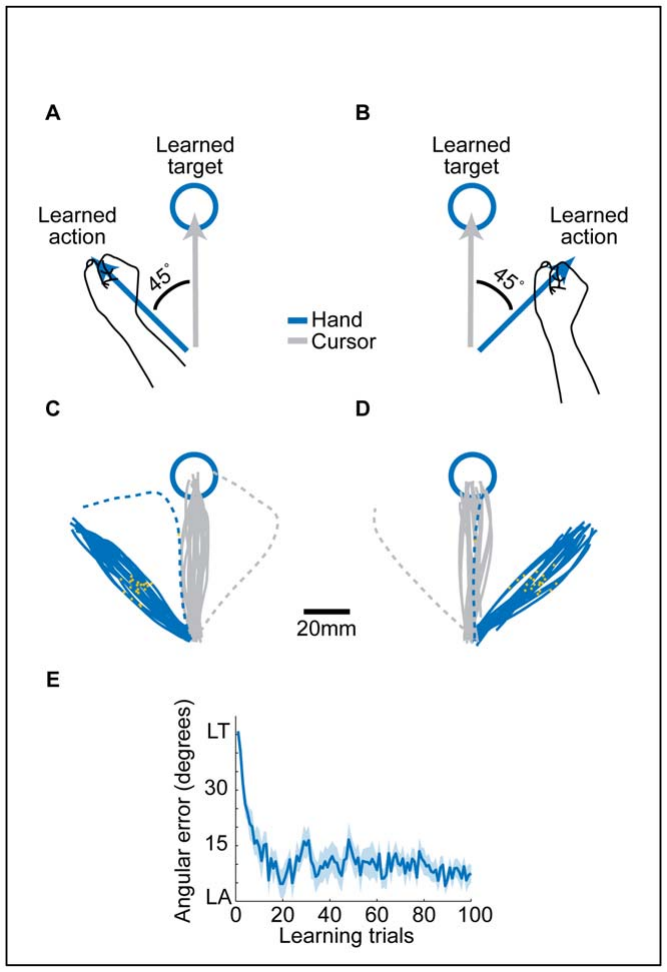
Block 2–The visuomotor rotation task. (**A–B**) In each learning trial, the subjects were required to perform hand movement in the direction of the learned action (blue arrows) in order to move a cursor (gray arrows) towards the learned target. The angular deviation of the cursor relative to the hand was 45°, either clockwise (A, 11 subjects) or counterclockwise (B, 11 subjects). Only one target location was used in all learning trials. (**C–D**) Trajectories of two subjects (exposed to either clockwise (C) or counterclockwise (D) rotation) in the first learning trial (dashed lines) and in trials 81–100 (solid lines). Orange dots represent the hand position 250 ms after movement onset. (**E**) Mean angular error (± SEM, N = 22 subjects) in the first 100 learning trials. Abbreviations: LT- learned target; LA- learned action.

In all 100 trials of the second block (“learning block”), the target (“learned target”) was presented at a single location. For one group of subjects (n = 11, Figure 3A) an angular deviation of 45° in the clockwise direction was applied to the hand-cursor relationship. Thus, in order to successfully reach the target, subjects needed to make a hand movement (“learned action”) in a direction that was 45° counterclockwise to the target. The experiment was mirror-flipped for a second group of subjects (n = 11, Figure 3B), where a counterclockwise deviation of 45° was applied. Figs. 3C and 3D depict single subjects' hand trajectories in the first learning trial (dashed blue lines) and in the last twenty trials (solid blue lines) of this block. The gray lines represent the cursor trajectories in these trials. The subjects learned the new rotation rule within approximately 20 trials, termed learning trials (Figure 3E).

**Figure 4:**
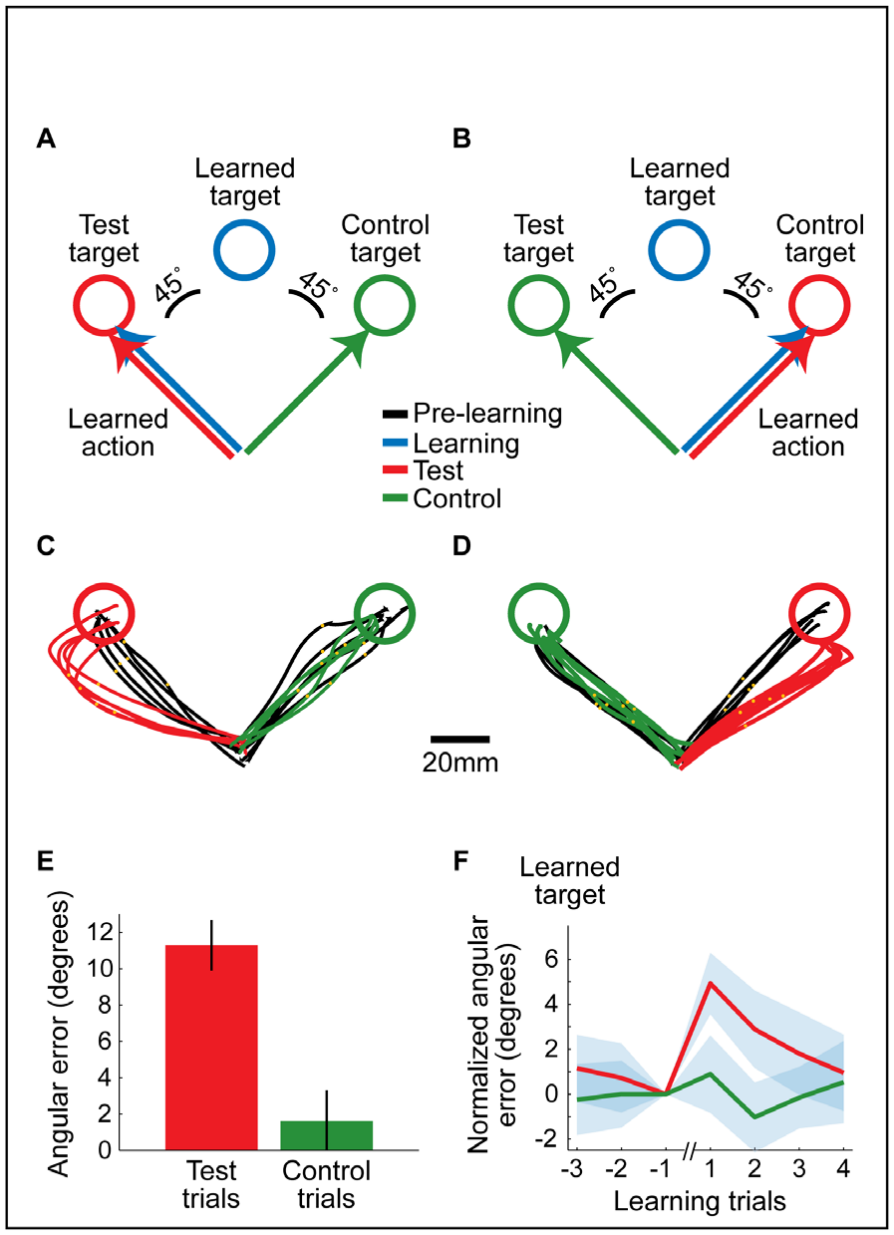
Block 3-Action-dependent learning. (**A–B**) Target locations (circles) and required hand movement directions (arrows) during the test block, for subjects who were exposed to clockwise rotation (A, 11 subjects) and counterclockwise rotation (B, 11 subjects). Note that the learned target and the test target required the same action (the learned action). (**C–D**) Single subjects' trajectories towards the test and the control targets during the test block (red and green, respectively), compared to trajectories towards these targets before learning (black). Orange dots represent the hand position 250 ms after movement onset. (**E**) Mean angular errors (± SEM, N = 22 subjects) in movements to the test (red) and control (green) targets during the test block. (**F**) Mean angular deviation (± SEM, N = 22 subjects) of hand movements in learning trials, before and after responses to the test target (red) and the control target (green). The errors are normalized to show the relative deviation from the preceding learning trial (trial number −1 on the x-axis).

In the third block (“test block”), subjects continued to move to the same learned target (presented with perturbation, as in the second block) but in some interleaved trials they were also presented with two other targets, counterclockwise (45°) and clockwise (−45°) to the learned target. To correctly reach these targets, subjects needed to make direct movements to the target location, just as they did in the first block, when no rotation was applied. For subjects who learned the clockwise perturbation, we defined the target at 45° as “test target” and the target at −45° as “control target” (Figure 4A, red and green, respectively). The definition of a target as a “test” or “control” was mirror-flipped for subjects who learned the counterclockwise perturbation (Figure 4B). This third block comprised 150 trials presented in a pseudorandom order, with every 30 trials containing one test target, one control target, and 28 learned targets. Note that the main difference between the test and control targets is that the hand movement required to reach the test target (Figure 4, A and B, red arrows) was identical to the learned action (Figure 4, A and B, blue arrows).

Figs. 4C and 4D depicts subjects' hand trajectories towards the test and control targets, performed before learning (black lines) and during the test block (red and green lines). In line with previous studies [9]–[11], we found that subjects' trajectories to the control targets (green) were not statistically different (Figure 4E, p = 0.12, n = 22) from those made in the first block, indicating little or no generalization of the learned visuomotor rotation. Surprisingly, subjects' trajectories to the test targets (red), were significantly different from those performed in the first block (Figure 4E, mean angular difference of 11.3°±1.4°, p<0.0001, n = 22), suggesting generalization of the learned rotation in this direction.

We next tested if the presentation of the test or control target affected subjects' performance on subsequent learning trials (Figure 4F). Control targets did not significantly affect performance of the learned action, as the angular errors in subsequent learning trials were unaffected (Figure 4F, green, 0.89°±1.7° p = 0.36 and −1.0°±1.5° p = 0.25, in the first and second subsequent learning trials, respectively). Following a test target, however, response to the learned target was changed significantly, showing a transient increase of the error in the first and second subsequent learning trials (Figure 4F, red, mean difference of 4.9°±1.4° p<0.0001 and 2.9°±1.7° p = 0.002, respectively).

## Discussion

The results shown in Figure 4C–E, demonstrate the considerable difference between subjects' responses to the test and control targets. Only hand trajectories to the test target were affected by learning. What could be the source of this unexpected discrepancy?

The effect of learning on movements to the test target was assessed carefully, using three different controls: First, to ensure that the effect is learning related, we compared the subjects' hand trajectories made before and after learning (Figure 4, C and D, black vs. red trajectories). Second, to rule out that the effect is related to the proximity of the test target to the learned target, we compared between hand trajectories to the test target and an equidistant control target (Figure 4, C and D, red and green trajectories). Third, to exclude the possibility that the effect is a result of spatial differences between the test and control targets, the experiment was mirror-flipped for half of the subjects (Figure 4, A and B), so that the test and control targets flipped their locations. Thus, the targets differ only in that the required response to the test target is very similar to the learned action (Figure 4, A and B, red and blue arrows), as opposed to the required response to the control target (Figure 4, A and B, green arrows).

The subjects were therefore not merely mapping the learned target to a new action; rather, it is plausible that they were employing a form action-dependent learning. Namely, in the second block, subjects saw that their “learned action” resulted in deviated motion of the cursor, towards the learned target. In the third block, when subjects saw a target that required movement *in the same direction* as the learned action, it is likely that they implicitly predicted that this hand movement would result in cursor motion to the learned target and not the test target. Thus, subjects made initial errors in trajectory to the test target. When the test target was counterclockwise to the learned target, hand movements were biased counterclockwise (Figure 4C), and when the test target was clockwise to the learned target, hand movements were biased clockwise (Figure 4D). The resultant generalization pattern is asymmetric about the learned target.

**Figure 5:**
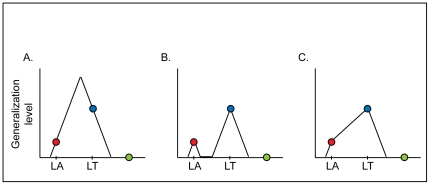
Alternative patterns of the generalization function. Stimulus-generalization is the transfer or “spreading” of a conditioned response to new stimuli. The extent of stimulus-generalization depends on proximity to the learned stimulus [1], [19]–[24]. For example, a pigeon that learned to peck as a response to a yellow stimulus (580 nm), will also peck, to some extent, as a response to yellowish stimuli (570 or 590 nm) but will not change its response to other colors. Generalization effects of visuomotor learning are commonly assessed by measuring after-effects in responses to stimuli that did not appear during learning [10], [11]. Previous studies on human subjects have found little or no generalization for stimuli located 45° or more away from the learned stimulus (this was termed “limited generalization” [10], [25]). Stimulus-generalization is assumed to be symmetrical about the learned stimulus [9]. Our results indicate that the pattern of the generalization function in visuomotor rotation learning is not symmetric about the learned stimulus. Movements to a target in the direction of the learned action were affected by learning, while movements to a target that was located at the same angular distance from the learned target were not affected. The generalization could still be symmetric (**A**), about a different direction, between the directions of the learned target and the learned action. However, it seems unlikely that the effect of visuomotor rotation learning would not be maximal in the direction of the learned target. Alternatively, the generalization function could be bimodal (**B**), with a peak in the direction of the learned target and a peak in the direction of the learned action. A third option is that the generalization function is unimodal, with relatively moderate slope in the directions between the learned target and the learned action (**C**). An experimental support for each of the last two options could be task-dependent: the whole range of hand movement directions (between the direction of the learned target and the direction of the learned action) is experienced during learning (Fig. 3E), with some directions experienced more than others. The visuomotor rotation task might therefore not be sensitive enough to examine the exact pattern of the generalization function. It should be emphasized that whether the generalization function is unimodal or not, whether it is symmetric or not, it depends not only in the spatial properties of the visual input, but also in the spatial properties of the motor output. Abbreviations: LA–learned action; LT–learned target.

Previous observations of generalization following learning of visuomotor rotation [1], [9], [10], including one from our laboratory [11], did not consider the effects of action-dependent learning. In these studies, the generalization of visuomotor adaptation across directions was assumed to be symmetrical about the learned target. Here we observed a specific adaptation effect in the direction of the learned action, indicating that the pattern of the generalization function is asymmetrical about the learned target (Fig. 5).

We also observed that subjects' responses to control and test targets differentially affected the trajectories of subsequent responses to the learned targets: test trials increased the error in subsequent learning trials while control trials did not (Figure 4F). This result, which suggests an action-dependent effect of interference to learning at the level of a single trial, further supports the involvement of an action-dependent learning component. Taken together, our main findings suggest that action-dependent effects can be observed in two aspects of learning: generalization and interference.

It is important to note that this study does not discriminate between the effects of actions per-se and their resultant sensations (e.g. proprioception) and/or reinforcements on adaptation. In this sense, we use the term “action-dependent learning” broadly.

The described action-dependent effects were isolated by testing a “chimeric” stimulus-action pair wherein the action (the learned action) was involved in learning, but the stimulus (the test target) was not. Based on our conceptual dissociation between action-dependent and stimulus-dependent learning components, the two were separated empirically. Our results are supported by two recent studies [12], [13] showing that repetition-induced movement biases occur simultaneously with error-based learning effects.

To conclude, this study dissociated sensory-motor learning into stimulus-dependent components (Figure 3E and Figure S1) and action-dependent components (Figure 4). In the context of the sensory-motor loop [2], [4], it is tempting to infer that stimulus-dependent effects reflect learning of the mapping of sensory inputs to motor outputs (How should I respond to this sensory stimulus?), while action-dependent effects reflect learning to map motor outputs to predicted sensory inputs (What happens when I execute this action?). Together, the stimulus-dependent and the action-dependent learning components enable a complete internal representation of the sensory-motor loop. It is possible that the two learning components originate from the same or different neural mechanisms; future physiological experiments may be instrumental for shedding light on this issue.

The ability to gain knowledge of the motor output is necessary to estimate the future state of the body in relation to the external world. Our findings support the notion that the brain predicts the sensory consequences of actions. The advantage of adaptivity of the sensory predictor is not necessarily specific to motor control. Recent studies have suggested that forward estimations occur in several brain areas, including the cerebellum [14], [15], the posterior parietal cortex [16], the vestibular system [17] and even the retina [18]. The generation of sensory predictions and the ability to modify them, in a changing but statistically predictable environment, appear to be a fundamental function of the nervous system.

## Materials and Methods

### 1. Ethics Statement

The experimental procedures were approved by the Hebrew University institutional review board. All subjects gave informed written consent prior to the experiment.

### 2. Subjects

Twenty two subjects (aged 20–27, 11 males 11 females) were paid to participate in the study. All subjects had normal or corrected to normal vision, were reported right-handed, had no reported neurological history, and considered as naïve subjects. Subjects were told that their payment depends on their performance level.

### 3. Behavioral task

Subjects were seated in a dark room and were asked to use their right hand in order to make reaching movements, using a lightweight robotic arm along a horizontal plane created by force boundaries (Phantom Haptic Interface, SensAble Devices, Cambridge, MA). A monitor projected a three-dimensional image of a cursor, which subjects controlled by moving the robotic arm, and a three-dimensional target through a mirror. Subjects positioned their head in front of the mirror and held the robotic arm. They could not see their arms or hands, but they were provided with the visual feedback of cursor position, which corresponded to their hand position. The positions of the cursor and of the robotic arm were sampled at 100 Hz by the device encoders and stored for off line analysis.

### 4. Trial flow

Subjects had to move a cursor with a 7.0 mm radius into a sphere with a 9.0 mm radius which appeared in the middle of the screen (‘center’). The subjects needed to keep the cursor inside the sphere until another sphere (the ‘target’), with a 10.0 mm radius, appeared, and the center disappeared. The subjects then had to move the cursor to the target within 900 ms. This relatively long interval allowed a limited but comfortable range of response time and movement time. Yet, subjects were instructed to perform fast and accurate movements. When the hand reached the target, the target changed its color. The cursor had to stay in the target for 300 ms, to consider the trial “successful”. A brief sound informed the subject of a success. A failure resulted in a different sound, which informed subjects that the trial was aborted. An inter-trial interval of 1.4 seconds separated the trials. All subjects read the instructions before the session has started and were tested verbally to confirm that the instructions were understood.

### 5. Trial types and session flow

There were three blocks of trials: pre-learning block, learning block and test block. The *pre-learning block* consisted of 144 *standard* trials. In each standard trial, the subjects had to move the cursor from the center to a target that appeared at one of eight locations (18 trials at each location). Targets were radially arrayed at a distance of 70.7 mm from the center, and were 45° apart (at 0°-rightward, 45°, 90°-forward, 135°, 180°-leftward, 225°, 270°-backward, and 315°, relative to the center).

The *learning block* consisted of 100 *learning trials*. The target in a learning trial (called the *learned target*) appeared always at the 90° location (forward to the center). In a learning trial, the relationship between hand movement and cursor movement was transformed; the location of the cursor was rotated 45° clockwise (group CW, 11 subjects) or 45° counter-clockwise (group CCW, 11 subjects) around the center, relative to the location of the hand. Therefore, in order to move the cursor to the *learned target*, the subjects had to make a hand movement at an angle of 45° from a direct path to the *learned target*: to the 135° location in group CW (Figure 3A) and to the 45° location in group CCW (Figure 3B). We called the required movement the *learned action*.

The *test block* immediately followed the learning block and consisted of 5 mini-blocks of 30 trials. Each mini-block contained 28 learning trials, 1 *test trial* and 1 *control trial* (Figure 4A and Figure 4B, for group CW and group CCW, respectively). The *test* and the *control* trials were standard trials with targets at the 45° and the 135° locations. The stimulus in a test trial (the *test target*) was located 45° away from the *learned target*, in the direction of the *learned action* (at the 135° location in group CW and at the 45° location in group CCW). The stimulus in a control trial (the *control target*) was also 45° away from the *learned target* (at the 45° location in the CW group and at the 135° location in the CCW group) but was not located in the direction of the *learned action*.

After the session, the all subjects were presented with a *post-learning block*, which was similar to the pre-learning block and consisted of 96 standard trials (12 trials at each of 8 locations). The visuomotor rotation was completely removed so that the learning could be “washed out” and after-effects on movements to the learned target could be measured (Figure S1).

Trials in the pre-learning block, in each mini-block of the test block and in the post-learning block were ordered pseudorandomly. The test target appeared before the control target in 53.6% of the mini-blocks. During the session, two breaks of 24 seconds enabled the subject to rest.

### 6. Data analysis

In each trial, the hand's direction was computed as the direction of the vector that connects the hand's position at movement onset to the hand's position 250 ms after movement onset.

Angular error in learning trials (Figure 3E) was calculated as the angular difference between the hand direction and the direction of the test target (the learned action).

The mean hand direction towards the test and control targets during the pre-learning block (computed for each subject separately) was used as a baseline to check learning-related changes in the third block. Thus, zero error in Figure 4E means that the hand direction in the third block was not changed, compared to the first block. There was no significant difference between the mean hand directions at the beginning and at the end of the pre-learning block.

In the pre-learning block, standard trials with a deviation of more than 20° (∼3 standard deviations of the mean hand direction made towards the test and control targets) between the hand's direction and the direction of the target were excluded. “Post-pre” comparisons of hand directions (Figure 4) also excluded angular differences of more than 20°. A more permissive criterion of excluding errors only when they were larger than 45° yielded similar results. Yet, the criterion of 20° was selected in order to restrict the analysis to trials in which we have a better estimate that the subject is more attentive and collaborating.

To check for the effect of test and control trials on learning trials (Figure 4F), we compared the hand trajectories in learning trials that appeared before and after each test or control trial, using the following normalization:

Norm_Err_(n)_  =  Err_(n)_ − Err_(−1)_, where Norm_Err is the normalized angular error (shown in Figure 4F) and Err_(n)_ is the measured angular error (as calculated in Figure 3E) at trial n, before (n<0) or after (n>0) the test/control trial. For example, if before a specific test/control trial the hand direction was 10 degrees away from the direction of the learned action (Err_(−1)_ = 10), and in the trial that followed the same test/control trial the error was 15 degrees (Err_(+1)_ = 15), then the normalized error for this one learning trial (Norm_Err_(+1)_) is +5 degrees (5 degrees larger than the error in the previous learning trial). Positive values of the normalized error mean larger error, and negative values–smaller error.

After-effects (Figure S1) were measured as the angular deviation of the hand's direction in the post-learning block, compared to the mean hand's direction in the pre-learning block.

Results are presented as means ± SEM. Paired Student's t-test was used to check for significance. Differences were considered significant if p<0.01.

## Supporting Information

Figure S1 **After-effects in movements to the learned target.**
(DOC)
