# Supplementary material for: Just Do It: Action-Dependent Learning Allows Sensory Prediction
Source: PLoS One. 2011 Oct 5;6(10):e26020. doi: 10.1371/journal.pone.0026020 (PMC3187836; doi:10.1371/journal.pone.0026020)
Supplement: Figure S1 — After-effects in movements to the learned target. (DOC) [file pone.0026020.s001.doc]

**Supporting Information**

**"**Just Do It: Action-Dependent Learning Allows Sensory Prediction**"**

**Itai Novick^1^ & Eilon Vaadia^1^**

^1^The Edmond and Lily Safra Center for Brain Sciences, Department of Medical Neurobiology, The Hebrew University of Jerusalem - Hadassah Medical School, Jerusalem 91120, Israel.

**Contents:**

- Figure S1. After-effects in movements to the learned target.
- Reference list for the supporting information.

**Supporting Information**

**Figure S1**. **After-effects in movements to the learned target.**

Hand movements towards the learned target were monitored before and after learning (in block 1 and immediately after block 3, respectively), when the hand-cursor relationship was not manipulated. In line with previous reports [1-3], when these movements were tested after learning, we observed significant aftereffects. These are shown by the mean angular deviation in the first three post-learning trials, of 29.4°±2.8° (p<0.0001), 19.2°±2.7° (p<0.0001) and 15.9°±2.6° (p<0.0001), respectively. Abbreviations: LA- learned action; LT- learned target.

**Reference List**

1. Krakauer, J. W., Ghilardi, M. F., and Ghez, C. (1999) Independent learning of internal models for kinematic and dynamic control of reaching. Nat.Neurosci. 2: 1026-1031.

2. Paz, R., Boraud, T., Natan, C., Bergman, H., and Vaadia, E. (2003) Preparatory activity in motor cortex reflects learning of local visuomotor skills. Nat.Neurosci. 6: 882-890.

3. Tseng, Y. W., Diedrichsen, J., Krakauer, J. W., Shadmehr, R., and Bastian, A. J. (2007) Sensory prediction errors drive cerebellum-dependent adaptation of reaching. J.Neurophysiol. 98: 54-62.
